# A Pharmacokinetics-Neural Mass Model (PK-NMM) for the Simulation of EEG Activity during Propofol Anesthesia

**DOI:** 10.1371/journal.pone.0145959

**Published:** 2015-12-31

**Authors:** Zhenhu Liang, Xuejing Duan, Cui Su, Logan Voss, Jamie Sleigh, Xiaoli Li

**Affiliations:** 1 Institute of Electrical Engineering, Yanshan University, Qinhuangdao, China; 2 Department of Anesthesia, Waikato Hospital, Hamilton, New Zealand; 3 State Key Laboratory of Cognitive Neuroscience and Learning & IDG/McGovern Institute for Brain Research, Beijing Normal University, Beijing, China; 4 Center for Collaboration and Innovation in Brain and Learning Sciences, Beijing Normal University, Beijing, China; Universiteit Gent, BELGIUM

## Abstract

Modeling the effects of anesthetic drugs on brain activity is very helpful in understanding anesthesia mechanisms. The aim of this study was to set up a combined model to relate actual drug levels to EEG dynamics and behavioral states during propofol-induced anesthesia. We proposed a new combined theoretical model based on a pharmacokinetics (PK) model and a neural mass model (NMM), which we termed PK-NMM—with the aim of simulating electroencephalogram (EEG) activity during propofol-induced general anesthesia. The PK model was used to derive propofol effect-site drug concentrations (*C*_*eff*_) based on the actual drug infusion regimen. The NMM model took *C*_*eff*_ as the control parameter to produce simulated EEG-like (sEEG) data. For comparison, we used real prefrontal EEG (rEEG) data of nine volunteers undergoing propofol anesthesia from a previous experiment. To see how well the sEEG could describe the dynamic changes of neural activity during anesthesia, the rEEG data and the sEEG data were compared with respect to: power-frequency plots; nonlinear exponent (permutation entropy (PE)); and bispectral SynchFastSlow (SFS) parameters. We found that the PK-NMM model was able to reproduce anesthesia EEG-like signals based on the estimated drug concentration and patients’ condition. The frequency spectrum indicated that the frequency power peak of the sEEG moved towards the low frequency band as anesthesia deepened. Different anesthetic states could be differentiated by the PE index. The correlation coefficient of PE was 0.80±0.13 (mean±standard deviation) between rEEG and sEEG for all subjects. Additionally, SFS could track the depth of anesthesia and the SFS of rEEG and sEEG were highly correlated with a correlation coefficient of 0.77±0.13. The PK-NMM model could simulate EEG activity and might be a useful tool for understanding the action of propofol on brain activity.

## Introduction

Understanding the mechanisms of action of general anesthetics in the central nervous system (CNS) may improve anesthetic drug administration and intra-operative monitoring. A few methods have been developed to explore anesthesia mechanisms. One is to use clinical or experimental observations to deduce the computation or communication mechanisms within the brain, including the analysis of physiological information obtained from monitor systems (such as the EEG), cerebral blood flow (CBF) and blood oxygenation level dependent (BOLD) signals [1–4]. Another approach is to interpret the mechanisms through computational modeling, using mathematical or physical theory to describe the brain’s inner workings, with varying degrees of physiological verisimilitude.

The electroencephalogram (EEG), as one of the oldest brain activity measurement methods, has been widely used in clinical experimental research [5, 6]. It is known that there are several distinguishing effects of the commonly used GABAergic (gamma-amino-butyric acid) anesthetic drugs on the EEG. In this study, we consider only the effects of propofol, which acts primarily on GABAergic type A (*GABA*_*A*_) receptors. First, during normal resting stages the spectral distribution of the EEG shows a strong suppression of alpha (8-13Hz) and beta (13-30Hz) power bands, and a dominance of slow wave delta/theta (0.5-8Hz) power [7]. Then, at low doses of propofol, the EEG shows signs of CNS excitation, with decreased oscillatory activity in slower frequency bands (3.5–12.5 Hz) and increased activity in the higher frontal beta frequency bands (12.5–25 Hz) [8, 9]. McCarthy et al. found that the interaction between the *GABA*_*A*_ current and an intrinsic slow potassium current (M-current) resulted in these phenomena [9]. Next, at deeper levels of anesthesia, the behavioral endpoints of sedation and unconsciousness emerge [10]. Loss of consciousness (LoC) is characterized by an increase in low-frequency EEG power (<1Hz), the loss of spatially coherent occipital alpha activity, and the emergence of a highly coherent frontal alpha rhythm [11–15]. It is suggested that the frontal alpha rhythm arises from propofol potentiating the strength of projections from the cortex to thalamus [14]. During the maintenance period of propofol-induced general anesthesia, the EEG spectrum is still dominated by low frequency activity but at a magnitude somewhat less than during induction. This rise and fall in low frequency power during anesthetic induction is often referred to as the “biphasic effect.” Steyn-Ross and colleagues proposed a mean-field model using the thermodynamic phase transition theory to characterize the abrupt change in cortical state from a highly activated equilibrium state to cortical suppression [16–19]. Finally, the burst suppression EEG pattern is seen in a very deep level of anesthesia, which is characterized by isoelectric periods interspersed with high amplitude activity [10]. A lot of etiologies can cause burst suppression besides general anesthesia, such as coma, stroke, head trauma, anoxia, early infantile encephalopathy, and hypothermia [10, 20–24]. Burst suppression is thought to occur through the interaction between neuronal dynamics and changes in cerebral metabolic rate of oxygen (CMRO) [25].

The models depicted above have successfully reproduced some features of the EEG under propofol-induced general anesthesia and have contributed to our understanding of anesthesia mechanisms. However, to the best of our knowledge, there has been no single model that could completely reproduce the simulated neural signals of the whole process of anesthesia in the macro scale. Also, few models characterizing EEG rhythms relate actual drug levels in a clinical setting to EEG activity. Thus, in this study we construct a model which takes the actual anesthetic concentration as a control parameter to derive the simulated anesthesia EEG. The intention is to bridge the gap between clinical and behavioral anesthetic manifestations and the underlying mechanisms of the anesthetic effects on the brain.

Pharmacokinetic (PK) modeling is used to describe how the concentration of a drug varies with time in the body, and pharmacodynamic (PD) modeling explains the relationship between drug tissue concentration and drug effect. The direct reflection of the effect of anesthesia is in neural activity. However, the PD model cannot generate EEG data, only an index of drug effect. This motivated us to build a new PKPD-like model to link up actual anesthetic drug concentration, effect-site concentration (*C*_*eff*_) and the EEG. Schnider's propofol PK model is selected due to its simplicity and wide inter-individual range [26]. The PD model is based on Steyn-Ross and Sleigh’s mean-field cortical model [18], which is derived from physiological and anatomical understanding of cortical connectivity; and has the advantage that its parameters are based on experimentally measured physiological quantities. In this study, the mean-field cortical model was simplified with assumptions; and empirical priors were used to emulate realistic signals. Our simplified model described the average activity of the cortex with two state variables, which summarized the behavior of millions of interacting neurons over time, and which we have termed the neural mass model (NMM). The advantage of this neural mass model is that it describes the macro phenomenon (EEG) based on the collection of micro neuronal activity and takes into account the pharmacological effect of anesthetic agents on neuronal ionic channels. This type of mesoscale model is suitable for comparison to EEG or electrocorticogram (ECoG) data, due to the fact that EEG electrodes measure the collective behavior of neuron population as a result of the large electrode spatial scale [27]. This model also has the advantage of significant literature displaying its applicability to modeling both anesthesia and coma, as well as other similar phenomena such as seizures and sleep [17, 18, 27–29]. In the following study, we name the constructed model a pharmacokinetics-neural mass model (PK-NMM).

Given the actual drug concentration and patient’s information, the combined model could produce simulated EEG-like (sEEG) data. To investigate how well the sEEG data behaved, we compared them with real EEG (rEEG) from the forehead during propofol-induced general anesthesia with respect to the frequency spectrum, nonlinear dynamics and high-order spectrum. There are some reasons for choosing these indices. Firstly, neural oscillations are a basic character of neuronal population activity, and the most intuitive to measure. So the typical short-time Fourier transform method was used to compute the EEG spectrogram. Secondly, population neural activity exhibits nonlinear behaviors, and there are many nonlinear methods to quantify these dynamic characteristics, such as Hurst exponent, detrended fluctuation analysis, entropy and others [30–33]. Also, a number of papers have shown that permutation entropy (PE) is able to quantify reliably the transition of the brain from the wake state to the state of general anesthesia [31, 34, 35]. So, PE is employed to evaluate the transient changes of rEEG and sEEG signals. In addition, various studies suggest that modulation of high and low frequency oscillations is an important mechanism during anesthesia [5, 36, 37]. Cross frequency coupling (CFC) plays a foundational role in identifying dynamical states during anesthesia. Bispectral analysis based on higher-order statistics is a typical CFC measure which can detect coupled nonlinear oscillators[38]. The bispectrum-derived SynchFastSlow (SFS) is sensitive to phase-phase coupling in different frequency bands and shows a robust correlation with loss of consciousness at the induction of propofol general anesthesia [39]. So, the SFS of the two types of signals were compared in this study to reveal the inter-frequency phase relations in the EEG.

This paper is organized as follows. Section 2 presents the details of the PK-NMM model, the calculation process of the sEEG, clinical experimentation and evaluation methods. The results are given in Section 3. Finally, the discussion and conclusion are given in Section 4.

## Materials and Methods

### Pharmacokinetics-neural mass model (PK-NMM)

#### Pharmacokinetics (PK) model

**Fig 1:**
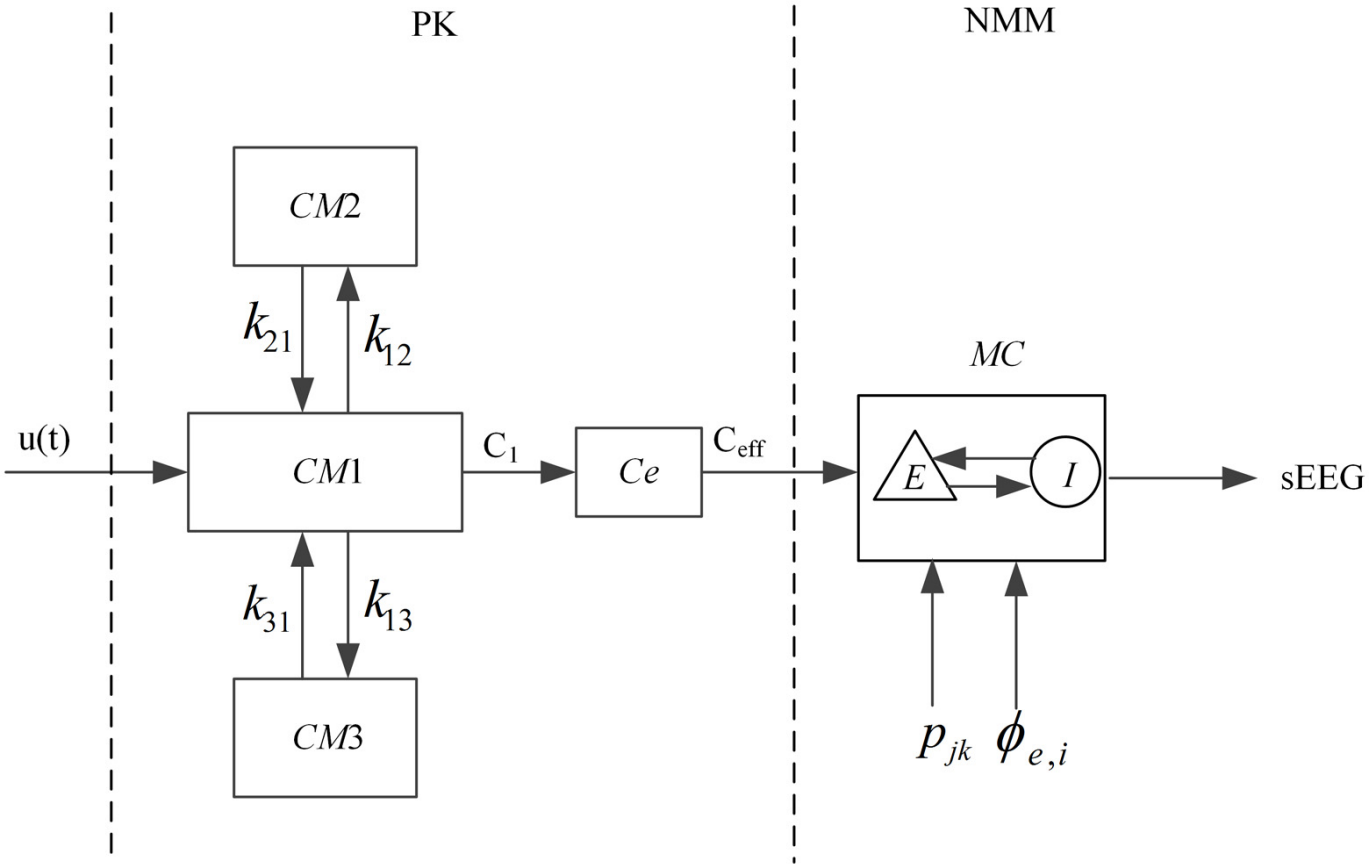
The graphical representation of the pharmacokinetics-neural mass model (PK-NMM). The compartments of the PK model and the drug transfer rate are shown, as well as the macrocolumn incorporated into the neural mass model.

Given a certain propofol drug delivery protocol, the Schnider PK model is used to obtain the effect-site drug concentration. Taking this effect-site concentration as the input of the neural mass model, the sEEG data are derived. A flow chart is drawn to illustrate the PK-NMM model, as shown in Fig 1.

The left part of Fig 1 describes the three-compartmental PK model, where *CM*1, *CM*2, and *CM*3 denote the central compartment and the other two peripheral compartments, respectively. *Ce* represents the effect compartment and *C*_*eff*_ is the propofol concentration at the effect compartment. *u*(*t*) is the infusion rate of propofol. The details of this PK module can be described as the following equations [26, 40]:

$$\left\{ \begin{array}{l} \frac{dC_{1}(t)}{dt}=-(k_{10}+k_{12}+k_{13})C_{1}(t)+k_{21}\frac{V_{2}}{V_{1}}C_{2}(t)+k_{31}\frac{V_{3}}{V_{1}}C_{3}(t)+\frac{1}{V_{1}}u(t)\\ \frac{dC_{2}(t)}{dt}=k_{12}\frac{V_{1}}{V_{2}}C_{1}(t)-k_{21}C_{2}(t)\\ \frac{dC_{3}(t)}{dt}=k_{13}\frac{V_{1}}{V_{3}}C_{1}(t)-k_{31}C_{3}(t) \end{array}\right.$$ (1)

$$\frac{dC_{eff}(t)}{dt}=k_{e0}(C_{1}(t)-C_{eff}(t))$$ (2)

where *C*_1_, *C*_2_ and *C*_3_ denote the drug concentration in the central compartment and the other two peripheral compartments, respectively. The constants *k*_*ij*_(*i*,*j* = 1,2,3,*i* ≠ *j*) indicate the drug amount transfer rate from the *i* th compartment to the *j* th compartment. The constants *V*_*i*_(*i* = 1,2,3) represent the volume of the *i* th compartment and *k*_e0_ reflects the transfer ratio between the central compartment and the effect compartment. In this module, some personalized parameters are determined with reference to [40], as shown in Table 1. In Table 1, parameters *C*_11_,*C*_12_ and *C*_13_ represent the clearance rates of the corresponding compartments, and the drug amount transfer rates are calculated according to the clearance rates:

$$k_{10}=\frac{C_{11}}{V_{1}},k_{12}=\frac{C_{12}}{V_{1}},k_{13}=\frac{C_{13}}{V_{1}},k_{21}=\frac{C_{12}}{V_{2}},k_{31}=\frac{C_{13}}{V_{3}}$$ (3)

In summary, the effect-site concentration of propofol is associated with patient’s age, weight, height and gender.

**Table 1:** The PK parameters of the Schnider propofol model.

PK parameters	Values or computational formulas
*V*_*1*_	4.27
*V*_*2*_	18.9–0.391*(age-53)
*V*_*3*_	238
*C*_*11*_[1/min]	1.89+0.0456*(weight-77)-0.0681*(1bm-59)+0.0264*(height-177)
*C*_*12*_[1/min]	1.29–0.024*(age-53)
*C*_*13*_ [1/min]	0.836
lbm (for male)	1.1*weight-128*weight^2^/height^2^
lbm(for female)	1.07*weight-148*weight^2^/height^2^

lbm represents the lean body mass

#### Neural mass model (NMM)

As described above, the cortical neural mass model is based on that of Steyn-Ross et al.[18] from an earlier model of the waking cortex[41, 42]. This model regards the brain as an active medium where all the cortical neurons are divided into interacting excitatory or inhibitory subpopulations. There are three connectivity types within a cortical macrocolumn—short range (intracortical), long-range (cortico-cortical) and exogenous (subcortical) connections. In the local area, excitatory and inhibitory populations interact with each other and themselves (short-range connections). The excitatory populations can also form long-range connections with excitatory and inhibitory populations across distant areas of the cortex. The right part of Fig 1 shows the sketch of the basic element of this neural mass model—a macrocolumn (*MC*), which is an assembly of about 100,000 correlated excitatory neurons (*E*) and inhibitory neurons (*I*). The ratio of excitatory to inhibitory neurons in the cortex is about 85%:15%. The cortex is modeled as a collection of macrocolumns. The symbol *p*_*jk*_ (*j*,*k*∈{*e*(*excitatory neuron*),*i*(*inhibitory neuron*)}) represents input from the subcortex. *ϕ*_*ee*_ and *ϕ*_*ei*_ represent *e*→*e*,*e*→*i* input from distant macrocolumns. Physically, the EEG is generated by the longitudinal current flowing along the apical dendrites of excitatory neurons which are aligned with an axial symmetry perpendicular to the cortical surface [43]. The potential due to the distributed current sources and sinks induced by afferent synaptic activity along these aligned excitatory dendrites can be approximated at the cortical surface by a dipole term [17]. The deviation from rest of the mean excitatory soma membrane potential ($h_{e}-h_{e}^{rest}$) has been demonstrated to be proportional to the sign-reversed image of the extracellular local field potential (LFP) [44]. Because the EEG is a spatially smoothed version of the LFP, it is reasonable to assume that it will be proportional to excitatory soma membrane potential (*h*_*e*_). In contrast, the dendrites and axons of those of the 15% inhibitory neurons orient at random with approximately spherical symmetry. So the equivalent dipole term of inhibitory neurons will be inconspicuously small; consequently it is generally believed that the scalp-measured EEG is generated by fluctuations in the spatially-averaged excitatory membrane potential [19]. Although the direct effect of inhibitory populations on the EEG is negligible, the inhibitory neurons play a crucial moderating role on the behavior of the excitatory population, so can not be ignored in any physiologically plausible description of cortical activity.

#### Incorporating action of propofol to the neural mass model

**Fig 2:**
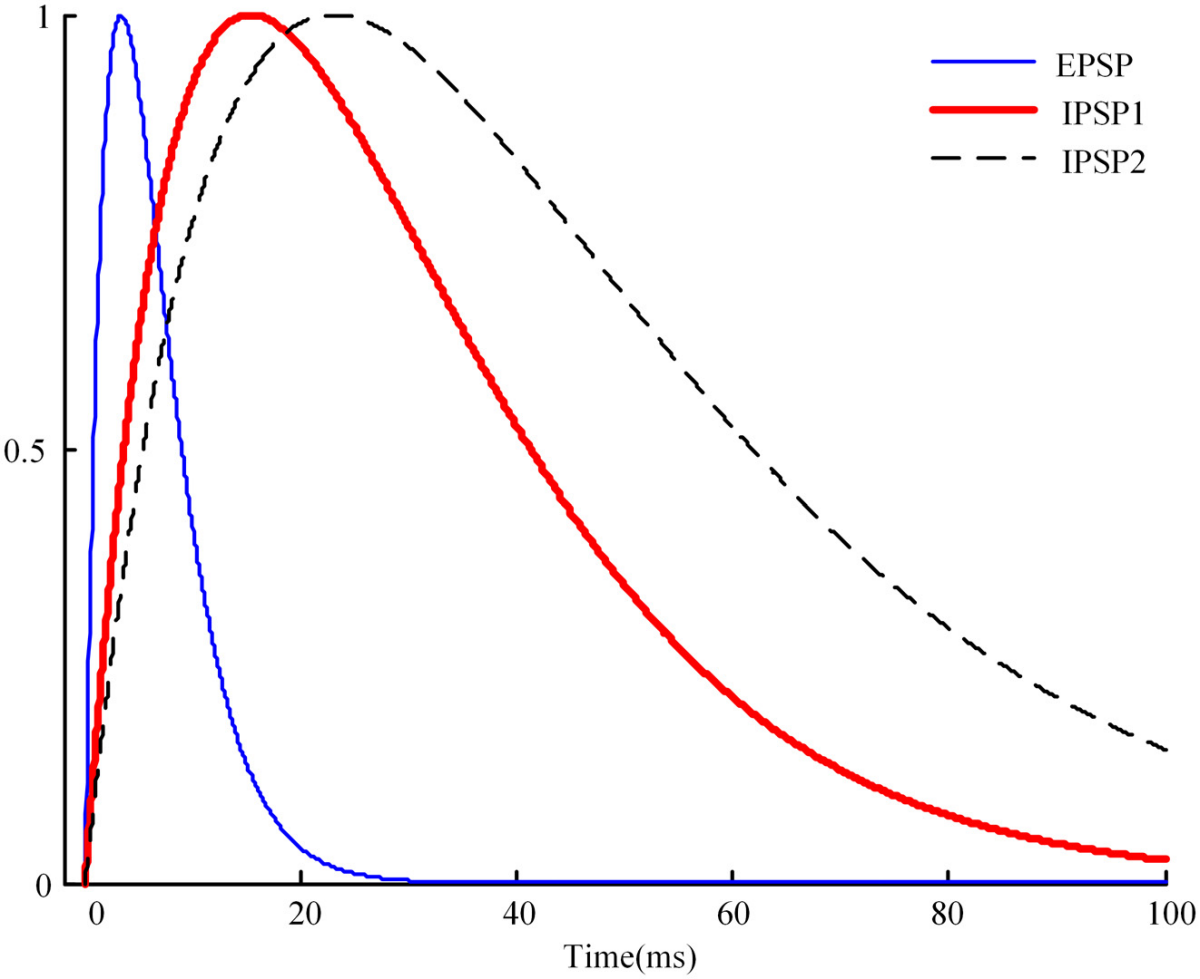
Time course for excitatory (blue curve), inhibitory (red curve), and anesthetic-modified inhibitory (dashed) postsynaptic potential. *λ* is the dimensionless anesthetic-effect scale factor giving the lengthening of the IPSP duration.

Assuming that a subject’s effect-site concentration of propofol has been derived using the Schnider PK model, a significant problem is how to apply this data to the neural mass model. To do this we must have some understanding of how anesthetic drugs affect brain function. So far, the most convincing mechanism for how commonly used GABAergic general anesthetic drugs operate at the cellular level is by enhancing the inhibitory effect of the GABA neurotransmitter, by keeping the chloride channels of the postsynaptic neurons open longer, allowing a larger negative charge to accumulate within the cell [45]. Liley et al. modeled the time course for the post synaptic potential (PSP) as a gamma-function impulse of the form *γt* exp(1−*γt*), where *γ* is the neurotransmitter rate constant for post synaptic potential (PSP)—*γ*_*e*_ for excitatory post synaptic potential (EPSP) and *γ*_*i*_ for inhibitory post synaptic potential (IPSP) [17]. The effect of the anesthetic drug propofol was incorporated into the model by lengthening the duration of IPSP by a dimensionless factor *λ*, which is done by replacing the IPSP neurotransmitter rate constant *γ*_*i*_ with *γ*_*i*_ */λ*. The time course for excitatory, inhibitory, and anesthetic-modified inhibitory postsynaptic potential is shown in Fig 2 [18]. IPSP duration increases with the increase of *γ*.

In the model of Steyn-Ross et al., the range of the input parameter *λ*—from the start of drug injection to the point of phase change into unconsciousness—varied linearly from 1.0 to ~1.5 (1.0 means no drug infusion). However, in reality the effect-site concentration of anesthetic drugs usually varies from 0 to some maximum value. As mentioned above, our aim is to apply effect-site drug concentrations to the NMM as the input. It is obvious that the range of input parameter *λ* of this model and the range of the real effect-site concentration do not match. So we made a linear transformation of *C*_*eff*_ to make the revised *C*_*eff*_(*rC*_*eff*_) change from 1.0 to ~1.49. Given an initial effect-site concentration series *C*_*eff*_, *rC*_*eff*_ was obtained by the following format:

$$rC_{eff}=\frac{0.49*C_{eff}}{\text{max}(C_{eff})}+1$$ (4)

The value of the term $\frac{0.49*C_{eff}}{\text{max}(C_{eff})}$ varies from 0 to 0.49, plus 1 will make *rC*_*eff*_ vary from 1 to 1.49. By making this transformation, the actual effect-site concentration of propofol was added to this NMM.

From the above description, we can see that the main idea of incorporating drug effect to the neural mass model was to use the moment-by-moment changes in drug effect-site concentration *C*_*eff*_ to determine the IPSP rate *γ*_*i*_. This was based on the widely accepted principles that propofol operates at the cellular level by enhancing the effect of GABA at inhibitory post-synaptic GABA_A_ receptors [45–47]; and that the drug effect on the EEG is primarily a consequence of its presence within the brain, modeled in this study as propofol effect-site concentration, *C*_*eff*_ [26].

#### Reproducing EEG time series

The macroscopic dynamics of neural activity in the cortex has been described by a set of non-linear continuum field equations [41]. In this study, these field equations were simplified to describe the behavior of the cortex in terms of parameters averaged over spatially localized populations of neurons. The primary variables of interest are the macrocolumn-averaged excitatory soma voltage *h*_*e*_ and inhibitory soma voltage *h*_*i*_. It is assumed that for certain ranges of anesthetic concentration during induction there exist steady states for *h*_*e*_ and *h*_*i*_. The random fluctuations of *h*_*e*_ (real-time *h*_*e*_) about its steady-state values are taken as the source of the scalp-measured EEG signal. That means that during anesthesia, the EEG signal is regarded as the difference between real-time *h*_*e*_ and steady-state *h*_*e*_.

The details of the equations are displayed in S1 File along with brief descriptions of the parameters and variables, as well as the calculation process of anesthesia EEG-like data. The model parameters are based on experimentally measured physiological quantities. A more in depth discussion of these equations can be found elsewhere [41, 42, 48].

### Drug and EEG recordings

#### Clinical protocol

In this study, the real anesthesia EEG data of nine volunteers (age 26 to 42, weight 59 to 120kg, three female) were taken from previously published work [49]. With the permission of the Waikato Hospital Ethical Committee, the volunteers (American Society of Anesthesiologists physical status I or II) were recruited to undergo a brief propofol anesthetic and recovered in accordance with normal procedures of the Australian and New Zealand College of Anesthesia (A.N.Z.C.A) guidelines. All subjects gave written informed consent after obtaining the permission of the hospital ethical committee.

#### Methods

**Fig 3:**
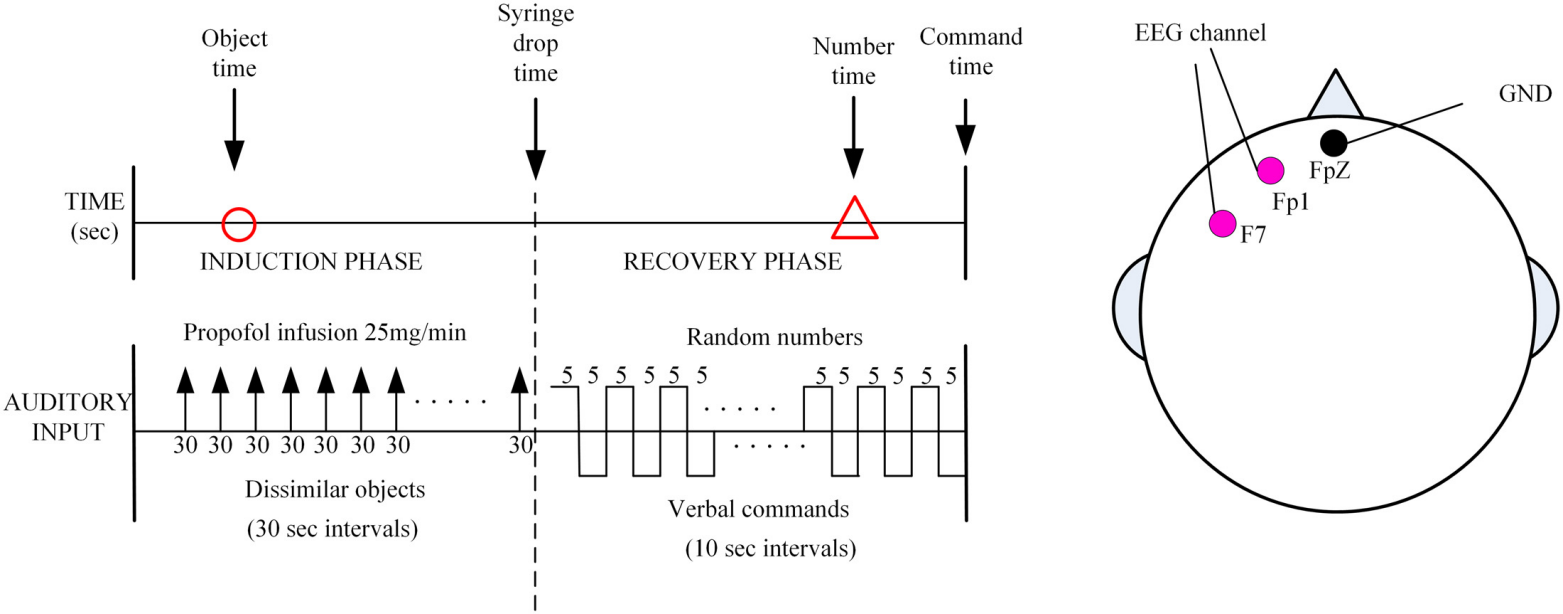
Diagram of the experimental design. The circle depicts the last object that could be recalled (the “object time”). The triangle depicts the time corresponding to the first number remembered during awakening (the “number time”).

In the work by Williams et al.[49], the authors performed an experiment in which nine fit human volunteers were given a brief propofol anesthetic to test conscious awareness in the absence of response to verbal command. As described more fully in this paper, prior to the surgery the volunteers were informed of the experimental protocol. Raw EEG recording was started at the beginning and lasted until the end of the experiment. The drug was infused at 150ml/h (1500mg/h) via a syringe driver. At the commencement of the infusion a verbal list of dissimilar objects was read to the participants at 30 second intervals; the time point of the last object they could remember during the induction was recorded as “object time”. The induction of anesthesia was ended when a syringe, filled with water, held between forefinger and thumb was dropped. This time point was recorded as “syringe-drop time”, and at this point the propofol infusion was ceased and the verbal list stopped. The participants were then allowed to recover and a pre-recorded tape of three-digit numbers and commands was started. The verbal commands were at 10-s intervals within the number sequences and consisted of simple commands such as “move your right foot”. The command itself lasted 5s. They were repeated for each of four limbs in a random fashion. The time point that the participant was able to respond correctly was the “command time”, at which point the experiment was terminated. The participant was asked which number they could first recall. This was recorded and the time this presented in the number sequence was recorded as “number time”. The experimental sequence is shown in Fig 3. The silver-silver chloride scalp electrodes were placed at the position of Fp1-F7 according to the 10–20 international system to produce bipolar signals. The ground electrode was placed at FpZ. The Aspect A-1000 EEG monitor (Aspect Medical Systems, Natick, MA, USA) was used to collect the real EEG signal (The sampling frequency is 256 Hz). Then the real EEG was down sampled to 100Hz for use.

According to the above experimental sequence, “syringe-drop time” was used as an indicator of loss of consciousness (LoC), the “command time” was regarded as the time point of recovery of consciousness (RoC). In this study, the period before “object time” is considered as the conscious state; the period between “syringe-drop time” and “number time” is considered as the unconscious state and the period after “command time” is considered as the recovery state.

The information about age, gender, weight and height for all the subjects is listed in Table 2. The event times of all the subjects are shown in Table 3 [49]. Three subjects (#4, #6 and #7) could not recall any numbers on awaking.

**Table 2:** The individual information for all the subjects.

Subject	Gender	Age (year)	Weight (kg)	Height (cm)
#1	M	39	98	191
#2	M	26	78	179
#3	M	30	120	198
#4	F	26	81	178
#5	M	37	78	177
#6	M	27	95	183
#7	F	42	68	165
#8	M	36	89	176
#9	F	35	74	169

**Table 3:** The time events for each subject.

Subject	“Object time”	*“*Syringe-drop time”	“Number time”	“Command time”
#1	180	283	435	475
#2	90	357	639	649
#3	90	421	792	802
#4	30	454	*	650
#5	120	289	360	380
#6	180	433	*	588
#7	90	202	*	545
#8	30	401	822	832
#9	90	355	560	570

“Object time” = the time point of the last object remembered for the subject during the induction phase.

“Syringe-drop time” = the time point that the subject dropped the syringe, denoting the end the induction “Number time” = the time point of the first number remembered during awakening.

* Subjects who did not remember any number until responding to verbal command.

“Command time” = the time point corresponding to the subject’s correct response to verbal command.

Three subjects (#4, #6 and #7) could not recall any numbers on awaking.

### Evaluation and statistics methods

To see how well the sEEG data resembled the rEEG data and whether they could describe some features of anesthesia, we computed and compared the frequency spectrum, permutation entropy and SynchFastSlow of these two signals.

#### Frequency spectrum

From the frequency spectrum, we could see directly how the frequency content and power changed with time. The MATLAB function *spectrogram* was used to compute the EEG frequency spectrum [50]. Then 10-second time series during conscious, unconscious and recovery states were extracted from the rEEG and the sEEG. The power spectra of these time periods were computed by using the short-time Fourier transform method. From the power spectra we can see the distribution of the two kinds of data over the frequency components during different anesthesia states.

#### Permutation entropy

Permutation entropy provides a simple and robust method to estimate complexity of time series, taking the temporal order of the values into account [31]. Furthermore, permutation entropy can be used as a measure of anesthetic drug effect [34] and as a means to detect different anesthesia states. The detailed algorithm can be found in [51]. We summarize as follows: first, a scalar time series {*x*_*1*_,*x*_*2*_,…,_*i*_,…,*x*_*t*_} is transformed to an *m*-dimension vector *X*_*i*_ = [*x*(*i*),*x*(*i*+*τ*),…,*x*(*i*+(*m*−1)*τ*] with the embedding dimension *m* and lag *τ*. Then, *X*_*i*_can be arranged in an increasing order. For *m* distinct numbers, there will be *m*! permutations. For a permutation with number *π*, let *f*(*π*) represents its frequency in all permutations. And the probability of the permutation is *p*_*i*_(*π*) = *f*(*π*)/(*M*−(*m*−1)*τ*). Finally, the permutation entropy for the time series is defined as follows:

$$H_{p}(m)=-\sum_{i=1}^{N-(m-1)\tau }p_{i}(\pi )\text{ln}p_{i}(\pi )$$ (5)

The corresponding normalized entropy:

$$0\leq PE=H_{p}(m)/\text{ln}(m!)\leq 1$$ (6)

#### SynchFastSlow

The SFS derived from the bispectrum was used as an index to describe the characteristics of cross frequency coupling (CFC) and can also be used to measure the depth of anesthesia. The steps of SFS calculation is summarized as follows:

For a digital epoch *x*(*i*), generate complex spectral values *X*(*f*) using FFT. For each possible frequency triplet, the bispectrum is defined as

$$B(f_{1},f_{2})=\left|X(f_{1})\cdot X(f_{2})\cdot X^{*}(f_{1}+f_{2})\right|$$ (7)

The complex SFS is defined as the log ratio of the sum of all bispectral peaks in the area from 0.5 to 47Hz over the sum of bispectrum in the area 40–47 Hz:

$$SychFastSlow={\text{log}}_{10}\frac{\Sigma_{\Omega_{fast}}B(f_{1},f_{2})}{\Sigma_{\Omega_{all}}B(f_{1},f_{2})}$$ (8)

where

$$\Omega_{fast}\equiv \left\{f_{1},f_{2}|f_{1}>0,f_{2}>f_{1},f_{1}+f_{2}\leq 47Hz\right\}$$

$$\Omega_{all}\equiv \{f_{1},f_{2}|f_{1}>0,f_{2}>f_{1},f_{1}+f_{2}\in [40,47Hz]\}.$$ (9)

The detailed algorithm can be found in [5, 39].

#### Correlation coefficient

The correlation coefficient was used to evaluate the degree of linear dependence of PE and the SFS index between the rEEG and sEEG signals. The correlation coefficient is calculated by

$$R(x_{1},x_{2})=\frac{C(x_{1},x_{2})}{\sqrt{C(x_{1},x_{2})\cdot C(x_{1},x_{2})}}$$ (10)

where, *x*_1_and *x*_2_are the indices derived from rEEG and sEEG, respectively. *C*(*x*_1_,*x*_2_) = *E*[(*x*_1_−*u*_1_)(*x*_2_−*u*_2_)] is the covariance, *E* is the mathematical expectation, *u*_*i*_ = *E*[*x*_*i*_],*i* = 1,2.

## Results

**Fig 4:**
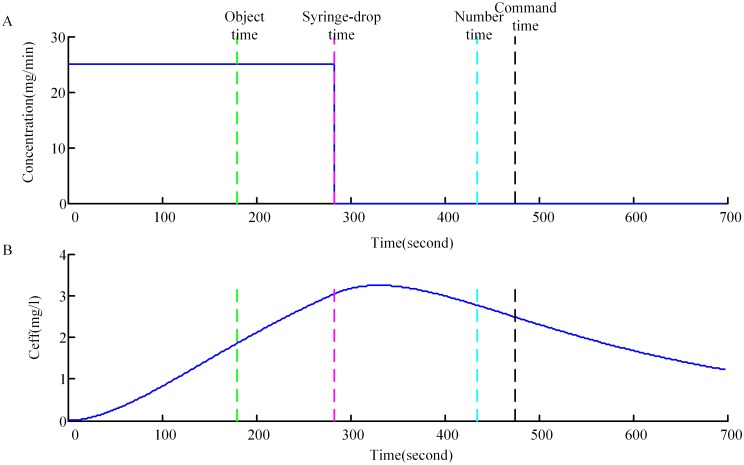
Results from propofol infusion of a single representative subject. (A) Time course of propofol infusion. The propofol infusion rate is 25mg/min. (B) Time course of the propofol concentration at the effect site obtained from the PK model for the same subject. “object time”, “syringe-drop time”, “number time”, and “command time” are marked with dashed lines.

The experimental protocol has been described in detail above, and Fig 4(A) gives a graphical representation from a single subject, where “object time”, “syringe-drop time”, “number time”, and “command time” are marked. Fig 4(B) shows the corresponding effect-site concentration (*C*_*eff*_) derived from the PK model. The *C*_*eff*_ values increased nonlinearly during induction and reached the maximum near the cessation of induction, then decreased soon after cessation.

**Fig 5:**
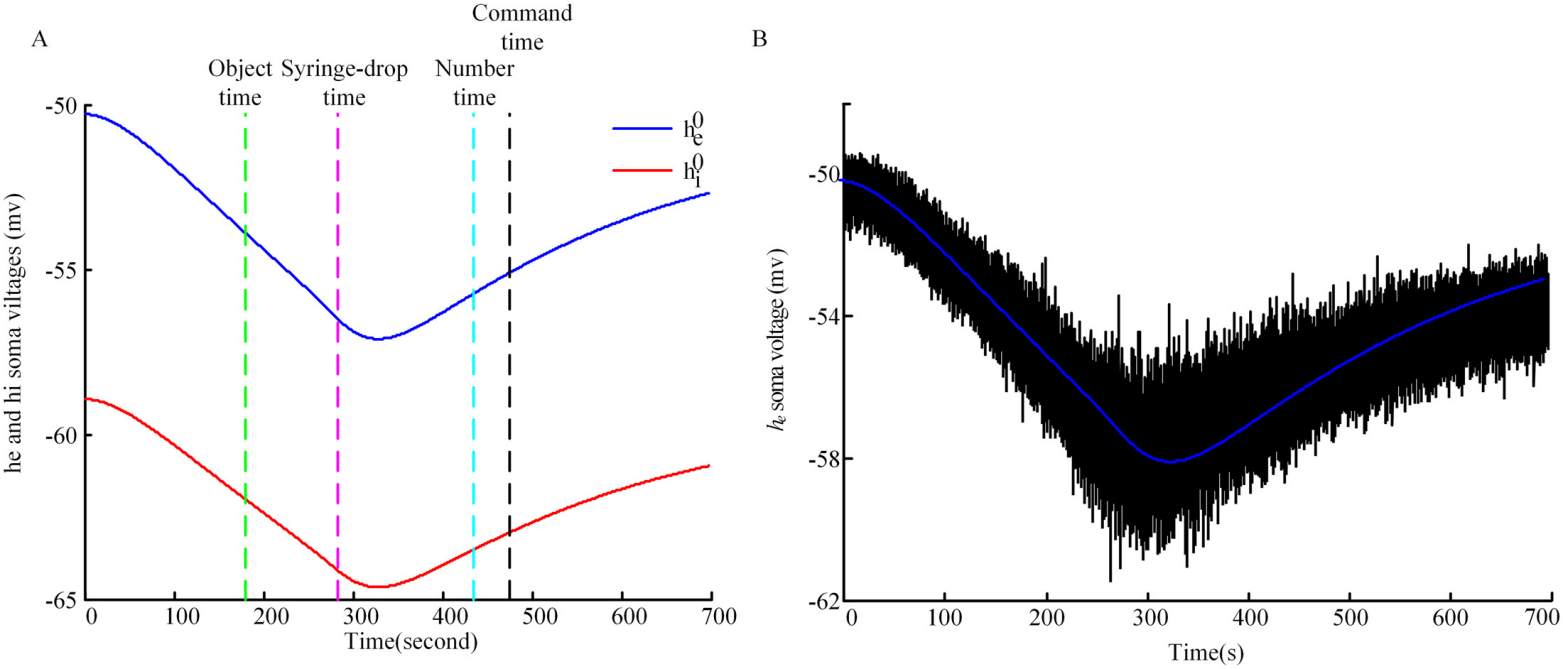
Computational processing of simulated EEG. (A) Model predictions for the stationary states for *h*_*e*_ (blue line) and *h*_*i*_ (red line). The superscript 0 in the legend represents stationary states. (B) The fluctuations of real-time *h*_*e*_. The blue line in between is the stationary values for *h*_*e*_. sEEG is calculated by using real-time *h*_*e*_ minus the values of *h*_*e*_ at stationary states. The fluctuations are displayed at 100*actual-size, and the line in between is the stationary state for excitatory neurons. The event time points are marked by dashed lines.

As previously described, the random fluctuations of excitatory neurons about their steady-state values are taken as the source of the scalp-measured EEG signal. Fig 5(A) shows the steady-state values for excitatory neurons (blue line) and inhibitory neurons (red line) for the same subject as used in Fig 4. The fluctuations of excitatory neurons along the steady-state curve are shown in Fig 5(B). In order to make the fluctuations visible, the amplitude is zoomed in 100 times, and the line in between is the stationary state for excitatory neurons.

**Fig 6:**
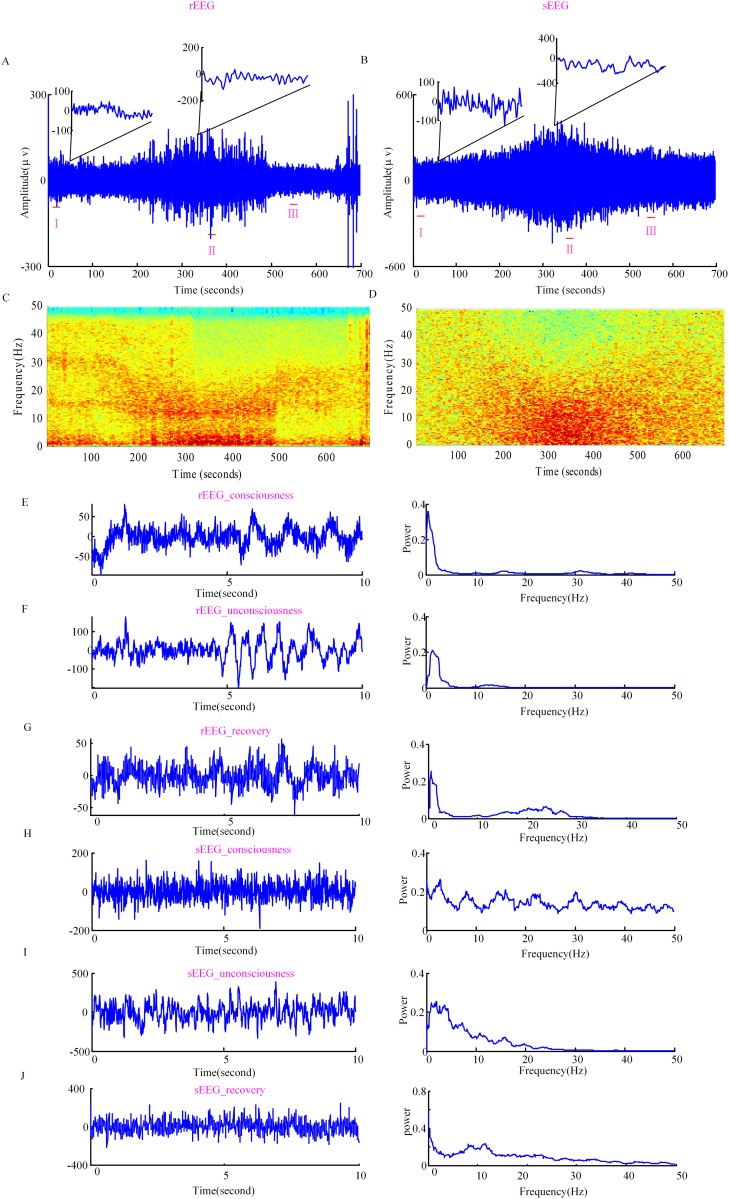
Real EEG and simulated EEG. (A) rEEG time series and (B) sEEG time series for a single subject. (C) and (D) show the EEG frequency spectrum of the two EEG signals. The dark red color denotes higher power and the blue color denotes lower power. (E), (F) and (G) show rEEG series of 10 s during consciousness (rEEG_consciousness), unconsciousness (rEEG_unconsciousness), recovery (rEEG_recovery) and the corresponding power spectra, respectively. (H), (I) and (J) show sEEG of 10 s during consciousness (sEEG_consciousness), unconsciousness (sEEG_unconsciousness), recovery (sEEG_recovery) and the corresponding power spectra, respectively. The 10 s EEG epochs extracted are labeled as I,II, III in the integral signal.

The simulated anesthesia EEG-like signal is calculated by subtracting the steady-state values of excitatory neurons at each time point from the curve of fluctuations of excitatory neurons along the steady state. Fig 6(A) and 6(B) show rEEG and sEEG for the same subject as a function of time. The upper portion of (A) and (B) are the expanded EEG waveforms of 1s in the conscious and unconscious states. It is shown that the electroencephalographic data during unconsciousness is more regular than during conscious state. To reveal the frequency content changes of the two types of anesthesia EEG signals, the frequency spectra are calculated, as shown in Fig 6(C) and 6(D). It can be seen that the sEEG spectrogram illustrates a change in the dominant EEG frequency pattern with the deepening of anesthesia, from high to low frequency; which is similar to the real EEG signal. However, the rEEG activity shows two prominent rhythmic activities around the delta frequency band (<3Hz) and the alpha frequency band at the start of LoC (the bifurcation between the delta frequency band and the alpha frequency band), lasting to the unconscious state. These two rhythmic peaks are not seen in the sEEG. Three 10 s EEG epochs are extracted, label as I(conscious EEG), II(unconscious EEG), III(recovery EEG) in Fig 6(A) and 6(B). Fig 6(E) shows real EEG of 10 s along with the power spectrum from one subject during consciousness. Fig 6(F) shows real EEG of 10 s along with the power spectrum during unconsciousness. The real EEG series of 10 s during recovery and the corresponding power spectrum are shown in Fig 6(G). Fig 6(H), 6(I) and 6(J) show simulated EEG series of 10 s and their corresponding power spectra during conscious, unconscious and recovery states, respectively. It is seen that during consciousness, for rEEG, the oscillation activity in low frequency bands (<5Hz) is strong, while the sEEG presents strong activity in 0~47 Hz. During unconsciousness the experimentally observed increases in low-frequency (<5Hz) power and more pronounced alpha oscillations are visible in the simulated series as well, but the theta activity still remains strong. During recovery state the increases in beta frequency power is seen in sEEG. The incomplete understanding of the physiological and anatomical structure of the cortex and the simplification of the spatial cortex all could lead to the differences seen with the experimentally observed data. From the variation of *C*_*eff*_ and the oscillation in the EEG, it seems that after drug infusion it takes some time before the EEG gives an obvious change. This is due to the fact that the anesthetic takes time to diffuse from the blood to the brain effect site, where the altered EEG response is generated.

**Fig 7:**
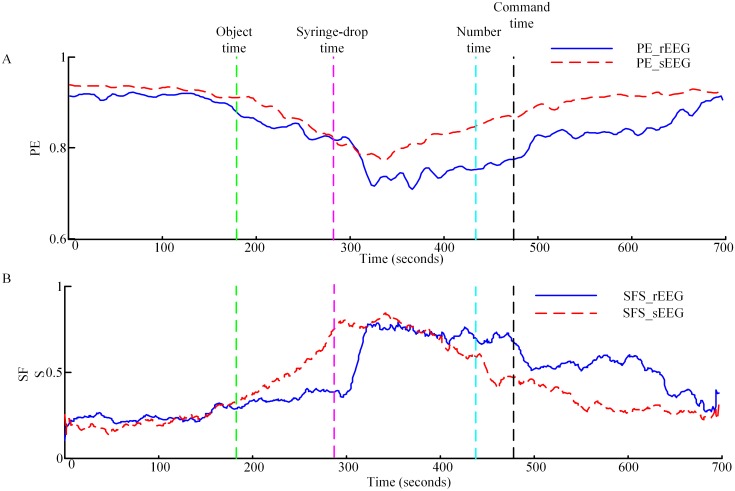
Permutation entropy and SynchFastSlow measures versus time. (A) Time course of PE with an embedding dimension *m* = 6 and lag *τ* = 1. The interval is 10 s and the overlapping size is 7.5s. The solid line represents the PE measure of rEEG (PE_rEEG) and dashed line represent the PE measure of sEEG (PE_sEEG), respectively. (B) Time course of SynchFastSlow. SFS_rEEG represents the SynchFastSlow of rEEG signal and SFS_sEEG represents the SynchFastSlow of sEEG signal. The event time points are marked by dashed lines.

The normalized PE and SFS were computed to compare the nonlinear dynamics of the sEEG and rEEG and to see if they could differentiate different anesthesia states based on the simulated EEG data. Fig 7 shows the PE and normalized SFS curves of rEEG and sEEG. For PE calculation, we selected *m* = 6 and *τ* = 1 [52]. Fig 7(A) shows the PE index of the two EEG signals for one subject. It is clear that the two curves have a similar trend. The PE values decrease with infusion of drug, reach the minimum near the end of drug cessation, then rise afterwards. It can be seen that PE applied to the sEEG successfully differentiates anesthesia states. The correlation coefficient between PE calculated from the sEEG and rEEG for all subjects averaged 0.80±0.13 (M±SD). The SFS curves of the same subject are shown in Fig 7(B). The curves reveal that with infusion of drug SFS values increase until injection stops, then the values decline slowly. The correlation coefficient of SFS calculated from the two EEG signals for all subjects is 0.77±0.13 (M±SD). From the curves we find that different anesthesia states could also be detected based on SFS of the sEEG.

**Fig 8:**
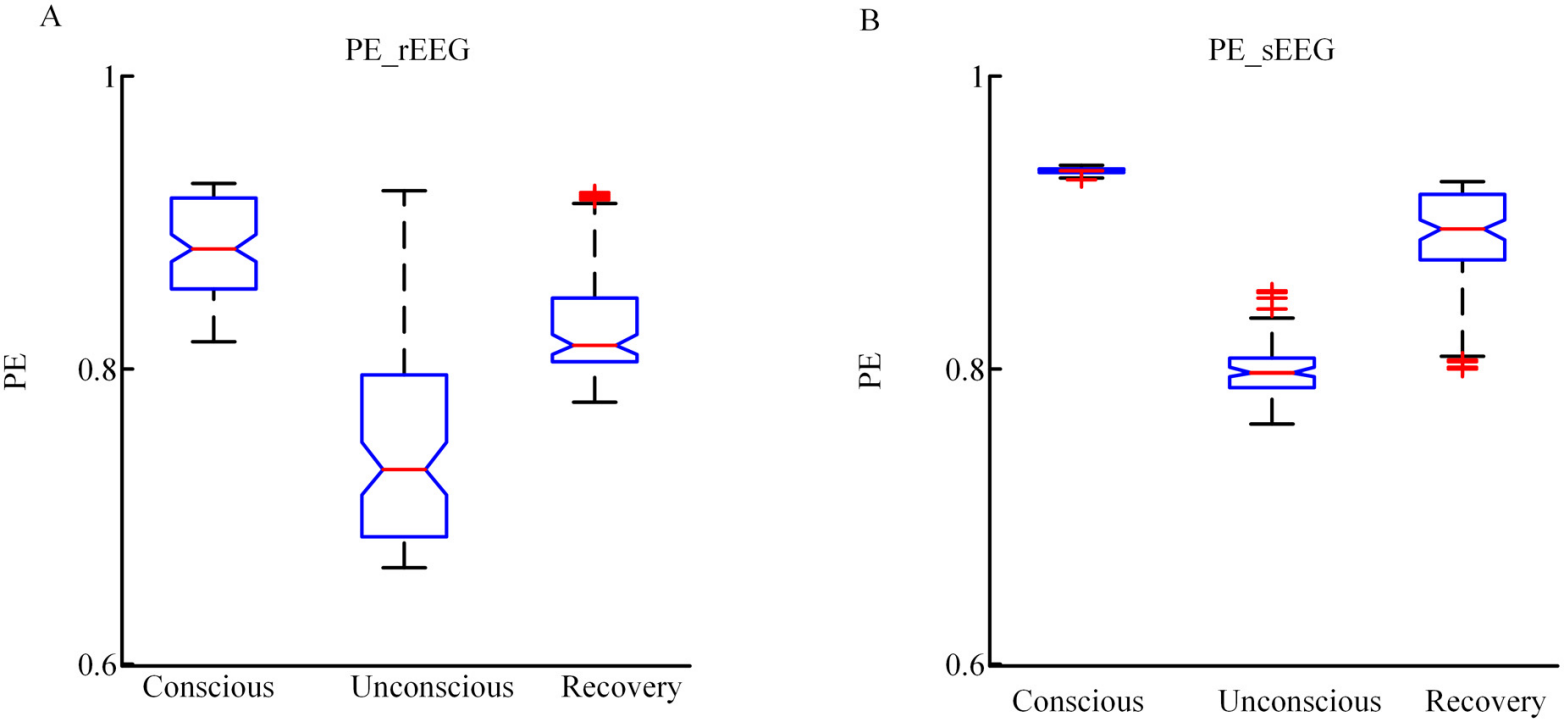
Boxplot of PE for rEEG and sEEG. (A) and (B) are boxplots for rEEG and sEEG at conscious, unconscious and RoC states, respectively.

Further, to compare the changes in PE for the sEEG and rEEG as anesthesia changes, PE values at the conscious state, unconscious state and recovery state were analyzed for each patient, and a box plot was constructed in Fig 8. The statistics of PE values of rEEG for all subjects are 0.88(0.82–0.93), 0.73(0.67–0.92), and 0.81(0.78–0.92) (median(min-max)) in the three states, respectively; the corresponding PE values of the sEEG were 0.93(0.93–0.94), 0.80(0.76–0.85), and 0.89(0.80–0.93) (median(min-max)). It can be seen that sEEG could reflect the cerebral dynamics during propofol-induced general anesthesia.

**Fig 9:**
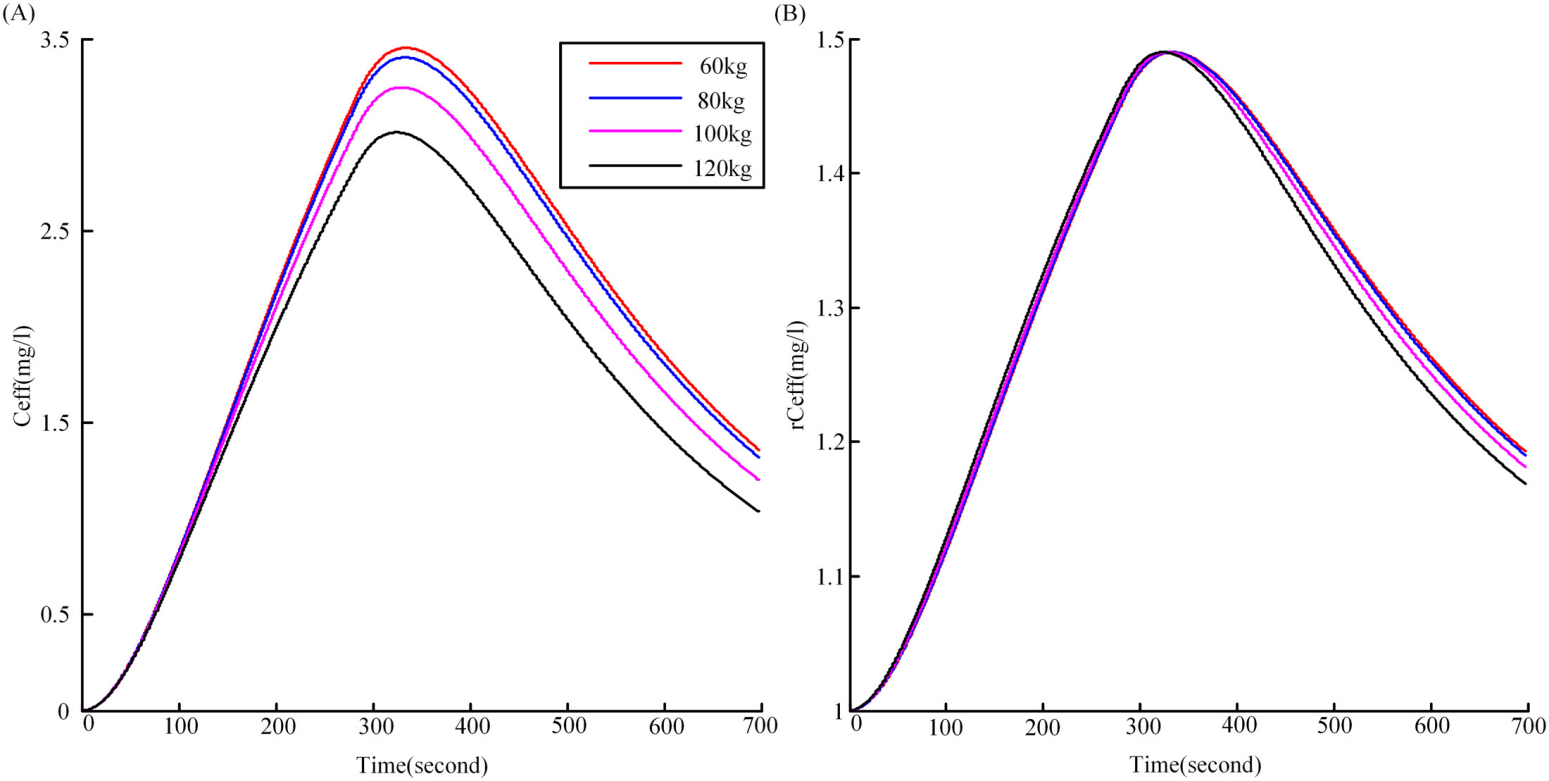
The effect-site concentration of propofol for one subject under four weights. (A) is the *C*_*eff*_ for one single subject under four weights (60, 80, 100, 120kg), (B) is the corresponding *rC*_*eff*_ under the four weights.

Another important issue that needs to be addressed is the effect of inter-subject variations (weight, age, height etc) on the EEG. Consider weight, four weights (60, 80, 100, 120 kg) were selected for each subject. The *C*_*eff*_ figures, just as for Fig 4(B), but now under the four weights for one single subject were shown in Fig 9(A). It can be seen from Fig 9(A), the *C*_*eff*_ showed differences under the four weights. As described earlier, to make the values of *C*_*eff*_ correspond with the input range of the NMM model, we made a transformation (eq 4) to give the *rC*_*eff*_, which was the real input of the NMM model. The *rC*_*eff*_ figures under the four weights for the same subject were shown in Fig 9(B). It is observed that the figures in (A) now collapse basically on one "normalized" figure (B). So the simulated EEG under the four weights would not show much difference, which meant that this procedure removed the EEG dependence on the weight.

**Fig 10:**
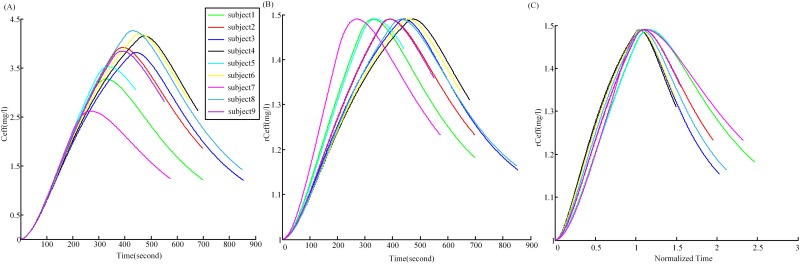
The effect-site concentration of propofol for all nine subjects. (A) is the *C*_*eff*_ for all subjects depending on time, (B) is the corresponding *rC*_*eff*_, and (C) represents the *rC*_*eff*_ with the time divided by their respective syringe-drop time.

On the other hand, the *C*_*eff*_ and *rC*_*eff*_ figures for all nine subjects were displayed in Fig 10(A) and 10(B), respectively. Different subjects had different reactions to propofol, with various peak time and peak *C*_*eff*_ (Fig 10(A)). But each of the subjects underwent the same states (awake, unconscious, recovery) during the experiment. Fig 10(C) presented a “normalization” of Fig 10(B) where the time for each figure was divided by their respective syringe-drop time, so that at the start of the unconscious state (at syringe-drop time) in normalized time is 1. It can be seen that the wide variety of figures in (A) fall basically on one "normalized" figure (C), close to normalized time 1, where they all had the value 1.49. And in some sense we were also normalizing the experimental recordings, because we were triggering on the same events (syringe drop, etc) for each subject, and so clearly the time was normalized in the same way, and furthermore dropping the syringe should mean "functionally similar" anaesthesia concentration in the brain. So the experimental and our theoretical procedure both basically eliminated the inter-subject variation dependence.

## Discussion and Conclusion

The mechanism of anesthesia is a hot topic of interest in the field of neuroscience. In this study, a combined model, which we have called PK-NMM, was constructed to simulate EEG activity during propofol-induced general anesthesia. The results showed that the PK-NMM model was able to reproduce EEG-like time series during propofol-induced general anesthesia. The performance of the sEEG in terms of the frequency spectrum, nonlinear dynamics and high order spectrum showed that sEEG could reflect many of the characteristics of the real EEG signal and reflect the cerebral dynamics during propofol-induced general anesthesia.

Several advantages can be concluded by analyzing this new model from theory to experiment. For the evaluation methods used to assess the performance of the sEEG, spectral distribution as well as nonlinear analysis methods was adopted. This practice represents a more comprehensive analysis of EEG data under general anesthesia. For the simulated EEG, different anesthesia states could be detected by using PE and SFS. Above all, most current anesthesia modeling strategies represent the target concentration of propofol in the neural population as a dimensionless factor that scales the potentiation of *GABA*_*A*_ inhibitory postsynaptic potentials[19, 25, 53, 54]. Vijayan et al. also considered the role of the hyperpolarization-activated current *I*_*h*_ besides the potentiation of *GABA*_*A*_in terms the actions of propofol [55]. McCarthy et al. modeled the action of propofol as the interaction between the *GABA*_*A*_ current and an intrinsic slow potassium current (M-current) in explaining the propofol-induced paradoxical excitation [9]. However, none of these incorporated actual drug concentration time-courses. In this study, the effect-site concentration of propofol was used as the determinant of the inhibitory PSP neurotransmitter rate constant of the neural mass model. Thus, the PK-NMM model established the relationship between clinical medication and anesthetic effects on the brain.

We test the performance of the model by analyzing the simulated data from different perspectives. For the simulated EEG of our model, the relatively good agreement with the real EEG (in terms of frequency spectrum, PE and SFS) indicates that this modeling approach is possible to be realized and could be a prospective way to reproduce the whole-time EEG. However, some limitations need to be addressed to improve this model in the future. First, the three-compartmental PK model gives a good approximation to the description of how the concentration of a drug varies with time in the tissue; however, this compartmental theory builds an abstract mathematical model to simulate the propofol distribution in the body based on rate of intravenous infusion, and thus does not reflect the effects of inter-individual variation in the real organ-specific physiology of drug distribution, metabolism and excretion [56, 57]. An alternative possibility would be to use a physiologically based pharmacokinetics model (PBPK) [57–60]. Second, the frequency spectrum of the sEEG does not show two prominent rhythmic peaks around the delta frequency band and the alpha frequency band. Although it is not precisely clear yet how these rhythms occur, there have been some studies to explain the underlying mechanisms leading to such phenomena. For instance, Hashemi et al. suggest that the alpha activity originates from the cortico-thalamic relay interaction, whereas the emergence of delta activity results from the full cortico-reticular-relay-cortical feedback loop with a prominent enforced thalamic reticular-relay interaction[61]. Ching et al. attribute frontal alpha rhythm to the enhancing of the strength of projections from the cortex to thalamus[14]. Alkire et al. propose that decreased excitation causes the firing mode of thalamic relay neurons to shift from a tonic to burst pattern, thereby producing delta activity [62]. These studies all validate that thalamocortical interactions play a critical role in producing the EEG rhythms during propofol anesthesia. But in our model subcortical sources were taken as the sum of a mean value plus a stochastic variation about the mean. We conclude that this discrepancy might be resolved through adding the effect of thalamocortical interactions. Third, the special anesthesia-induced burst suppression pattern [63, 64] is not considered in this study. Burst suppression is a unique EEG pattern seen in overdose of general anesthesia, and probably involves propofol inhibition of other intracellular metabolic processes, rather than positive modulation of the *GABA*_*A*_ receptor. Fourth, the experimental and our theoretical procedures both basically eliminate the inter-subject parameter (age, weight, gender etc) dependence (Figs 9 and 10). So the model is not able to predict small changes in EEG spectrum caused by inter-subject variations. In fact, it is certain that these parameters had some effect on the PK part of the model, and hence the propofol *C*_*eff*_ levels [26, 56, 65], but how these parameters affect the EEG remains largely unknown. The sources of inter-individual variance in EEG pattern are likely due to the large unexplained inter-individual differences in brain circuitry[66].

Besides, it is noteworthy that there are differences between the scalp-measured EEG and macro-columnar activity of neurons. In this study, we assume that the activity of macrocolumns can be regarded as the source of the scalp-measured EEG. There are some problems using the EEG measured at a single scalp location to reflect the activity of macrocolumns. The problem involves EEG source localization which aims to find the brain areas responsible for generation of the EEG waves. It consists of solving forward and inverse problems. Solving the forward problem starts from a given electrical source configuration representing the active neurons in the head. Then the potentials at the electrodes are calculated for this configuration. Dynamic models (e.g. our neural mass model) play an essential and complementary role as forward models that can be inverted given empirical data [67]. Thus, dynamic models are critical for integrating theory and experiments. The inverse problem attempts to find the electrical source that generates a measured EEG signal. To solve the inverse problem, repeated solutions of the forward problem for different source configurations as well as multichannel EEG signals are needed. Solving the problem of EEG source localization based on multichannel EEG signals will undoubtedly be a significant work. But, unfortunately, we only have a single frontal EEG channel, thus cannot derive more source information. In future modeling research, this needs to be addressed. For forward and inverse models, readers of interest can refer to [68, 69] for a review. That we use the activity of macrocolumns to describe the characteristics of the EEG is based on existing models which describe these characteristics based on a small set of neurons. For example, in the work by Hindriks et al.[54], the neural mean-field model models the dynamics of locally averaged membrane potential of different neuron types within the thalamocortical system during propofol anesthesia. The time series reproduced by this model are taken as simulated EEG and are compared with observed EEG. In the work by Ching et al.[14], the largest network studied considered 80 pyramidal (E) cells, 16 low threshold spiking (LTS) cells, 16 fast spiking (FS) cells, 6 thalamic reticular (RE) cells, and 6 thalamocortical relay (TC) cells, the interaction of which reproduced the propofol-induced alpha rhythm. In our study, we emphasize on showing that the model is able to reproduce some EEG phenomena.

In conclusion, the PK-NMM model reproduced EEG time series during propofol anesthesia. The simulated EEG time series could distinguish different anesthesia states, using standard EEG indices of depth of anesthesia.

## Supporting Information

S1 File The detailed description of the neural mass model and the computational process of EEG-like data. (PDF)
