# Supplementary material for: A Pharmacokinetics-Neural Mass Model (PK-NMM) for the Simulation of EEG Activity during Propofol Anesthesia
Source: PLoS One. 2015 Dec 31;10(12):e0145959. doi: 10.1371/journal.pone.0145959 (PMC4697853; doi:10.1371/journal.pone.0145959)
Supplement: S1 File — (PDF) [file pone.0145959.s001.pdf]

The detailed description of the neural mass model and the computational processing of EEG-like data are summarized below. The specific parameter meanings and their values are listed in Table A.

Table A. Symbol definitions and model constants

Symbol	Description	Value	Unit
$G_{e,i}$	peak amplitude of EPSP,IPSP	0.18, 0.37	*mv*
$\gamma_{e,i}$	neurotransmitter rate constant	0.3, 0.065	ms^{-1}
e	base of natural logarith	2.71828	
$h_{e,i}^{rest}$	cell resting potential	-70, -70	*mv*
$h_{e,i}^{rev}$	cell reversal potential	45, -90	*mv*
$N_{ee,ei}^{\beta}$	total number of local $e\rightarrow e, e\rightarrow i$ synaptic connections	3034,3034	
$N_{ie,ii}^{\beta}$	total number of local,$i\rightarrow e$, $i\rightarrow i$ synaptic connections	536,536	
$g_{e,i}$	sigmoid slope at inflection point	0.28, 0.14	mv^{-1}
S_e^{max}, S_i^{max}	maximum value for sigmoid function	1.1	ms^{-1}
$\tau_{e,i}$	membrane time constant	40, 40	*ms*
$\theta_{e,i}$	Inflexion-point voltage for sigmoid function	-60, -60	*mv*
Δt	timestep used in simulation	0.1	*ms*
$N_{ee,ei}^{\alpha}$	total number of synaptic connections from distant *e* populations	4000,2000	
$< p_{ee,ie} >$	exogenous(subcortical) spike input to e population	1.1,1.6	ms^{-1}
$< p_{ei,ii} >$	exogenous(subcortical) spike input to *i* population	1.6,1.1	ms^{-1}

The simulation parameters are chosen according to the nomenclature of Steyn-Ross et al. [1]

A schematic representation of the neural mass model is given by Figure A. Spatially averaged excitatory neurons (E) and inhibitory neurons (I) are taken into account to subsume the actual activity of cortical cells. The action played by excitatory neurons is marked with blue lines. There are three connectivity types within a cortical macrocolumn — short-range (intracortical) connection, long-range (cortico-cortical) connection and exogenous (subcortical) connection. The four $N_{jk}^{\beta} S_j$ ($j,k \in \{e(excitatory\ \ neuron), i(inhibitory\ \ neuron)\}$) are the short-range spike-rate contributions generated by within macrocolumn activity, the subscript jk means $j \rightarrow k$, i.e. type j acting on type k. The N_{jk}^{β} constants are the number of $j \rightarrow k$ local connections, and the S_j are their mean firing rates. The ϕ_{ee}, ϕ_{ei} are long-range excitatory spike-rate inputs from distant

macrocolumns elsewhere in the cortex, and they are expressed in the form of $\phi_{ee} = N^{\alpha}_{ee} S_e, \phi_{ei} = N^{\alpha}_{ei} S_e$. N^{α}_{ek} are the number of distant connections from excitatory populations. Only the excitatory populations are considered to form long-range connections to other macrocolumns. The four P_{jk} are inputs entering the macrocolumn from subcortical sources.

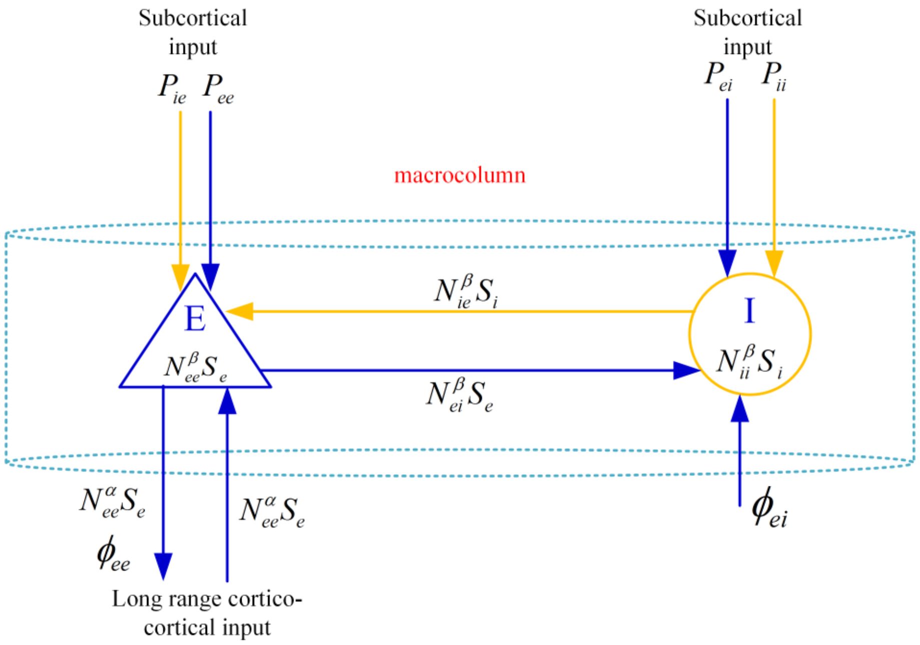

Figure A. A schematic illustration of the neural mass model. A macrocolumn with its excitatory neurons (E) and inhibitory neurons (I) are shown, as well as the short- and long- range cortical and extracortical connections.

The original nonlinear partial differential equations were simplified as two first-order ordinary differential equations by making three assumptions: the cortex is homogeneous; all inputs to the soma are regarded as fast variables that have already reached steady states before the soma can make a reaction; input to neuronal populations is added as the sum of a mean value plus a stochastic variation about a mean value.

The simplified equations are written as,

$$\frac{dh_e}{dt} = \frac{1}{\tau_e}[h_e^{rest} - h_e + \psi_{ee}(h_e) I_{ee}(h_e) + \psi_{ie}(h_e) I_{ie}(h_i)] \qquad (1)$$

$$\frac{dh_i}{dt} = \frac{1}{\tau_i}[h_i^{rest} - h_i + \psi_{ei}(h_i)I_{ei}(h_e) + \psi_{ii}(h_i)I_{ii}(h_i)] \tag{2}$$

Where h_e^{rest} and h_i^{rest} are the resting potentials for excitatory and inhibitory neurons, respectively. I_{jk} are the total j type cell voltage inputs to k type synapses. ψ_{jk} are dimensionless weighting factors for those voltage inputs, representing that excitation and inhibition are regulated by different ions and that the corresponding magnitude of the postsynaptic currents will depend on the active state of the neuron.

Each P_{jk} is written as its mean value ($< P_{jk} >$) plus a stochastic variation about the mean,

$$P_{jk} = < P_{jk} > + \sqrt{\alpha < P_{jk} >}\xi_n(t) \tag{3}$$

α is a dimensionless scale factor introduced to ensure that the stochastic fluctuations always remain small. Here we choose $\alpha = 0.01$. Each $\xi_n(t)$ (n=1,2,3,4) is an independent, Gaussian-distributed white-noise generator of zero mean and delta-function covariance.

I_{jk} and ψ_{jk} are expressed by:

$$I_{ee}(h_e) = \left[N_{ee}^{\beta} S_e(h_e) + N_{ee}^{\alpha} S_e(h_e) + < p_{ee} >\right] G_e e / \gamma_e \tag{4}$$

$$I_{ei}(h_e) = \left[N_{ei}^{\beta} S_e(h_e) + N_{ei}^{\alpha} S_e(h_e) + < p_{ei} >\right] G_e e / \gamma_e \tag{5}$$

$$I_{ie}(h_i) = \left[N_{ie}^{\beta} S_i(h_i) + < p_{ie} >\right] \lambda G_i e / \gamma_i \tag{6}$$

$$I_{ii}(h_i) = \left[N_{ii}^{\beta} S_i(h_i) + < p_{ii} >\right] \lambda G_i e / \gamma_i \tag{7}$$

$$\psi_{jk}(h_k) = \frac{h_j^{rev} - h_k}{\left|h_j^{rev} - h_k^{rest}\right|} \tag{8}$$

Where λ is the revised effect-site concentration of propofol (rC_{eff}). $S_e(h_e), S_i(h_i)$ are sigmoidal transfer functions that map from soma voltage to the output pulse rate (in pulses per second):

$$S_e(h_e) = \frac{S_e^{\max}}{1 + \exp[-g_e(h_e - \theta_e)]} \tag{9}$$

$$S_i(h_i) = \frac{S_i^{\max}}{1+\exp[-g_i(h_i-\theta_i)]} \tag{10}$$

By setting the derivatives to zero ($\frac{dh_e}{dt}=0, \frac{dh_i}{dt}=0$) and removing the noise ($\xi_n(t)=0$) in equations (1)-(2), the steady-state values of the excitatory and inhibitory neurons can be located by solving equations (1)-(10), as shown in Fig 5(A) in the main text. The steady state voltages for excitatory and inhibitory neurons are denoted as h_e^0 and h_i^0, respectively.

Substituting equations (3)-(10) to (1)-(2), equations (1) and (2) can be rewritten as the sum of drift terms and random noise terms [1],

$$\frac{dh_e}{dt} = F_1(h_e,h_i) + \Gamma_e(t) \tag{11}$$

$$\frac{dh_i}{dt} = F_2(h_e,h_i) + \Gamma_i(t) \tag{12}$$

where F_1, F_2 are drift terms,

$$F_1(h_e,h_i) = \frac{h_e^{rest} - h_e + \psi_{ee}\left[(N_{ee}^{\alpha}+N_{ee}^{\beta})S_e(h_e) + < p_{ee} >\right]\frac{G_e e}{\gamma_e} + \lambda\psi_{ie}\left[N_{ie}^{\beta}S_i(h_i) + < p_{ie} >\right]\frac{G_i e}{\gamma_i}}{\tau_e} \tag{13}$$

$$F_2(h_e,h_i) = \frac{h_i^{rest} - h_i + \psi_{ei}\left[(N_{ei}^{\alpha}+N_{ei}^{\beta})S_e(h_e) + < p_{ei} >\right]\frac{G_e e}{\gamma_e} + \lambda\psi_{ii}\left[N_{ii}^{\beta}S_i(h_i) + < p_{ii} >\right]\frac{G_i e}{\gamma_i}}{\tau_i} \tag{14}$$

Γ_e, Γ_i are random noise terms,

$$\Gamma_e(t) = \frac{\sqrt{\alpha}}{\tau_e}\left(\frac{\sqrt{< p_{ee} >}\xi_1(t)G_e e\psi_{ee}}{\gamma_e} + \frac{\sqrt{< p_{ie} >}\xi_2(t)G_i e\psi_{ie}\lambda}{\gamma_i}\right) \tag{15}$$

$$\Gamma_i(t) = \frac{\sqrt{\alpha}}{\tau_i}\left(\frac{\sqrt{< p_{ei} >}\xi_3(t)G_e e\psi_{ei}}{\gamma_e} + \frac{\sqrt{< p_{ii} >}\xi_4(t)G_i e\psi_{ii}\lambda}{\gamma_i}\right) \tag{16}$$

The above equations cannot be solved analytically. Then we consider numerical simulations of these equations. To perform time-series simulations, the Euler method was used to update each of the variables, the equations in simulated form are:

$$h_e^{n+1} = h_e^n + \Gamma_e^n\Delta t + \left[\frac{dh_e}{dt}\right]_{drift}\Delta t \tag{17}$$

$$h_i^{n+1} = h_i^n + \Gamma_i^n \Delta t + \left[\frac{dh_i}{dt}\right]_{drift} \Delta t \quad (18)$$

The drift components in equations (13) and (14) in simulated form become:

$$\left[\frac{dh_e}{dt}\right]_{drift} = \left[\frac{h_e^{rest} - h_e^n + \psi_{ee}\left[(N_{ee}^{\alpha} + N_{ee}^{\beta})S_e(h_e^n) + < p_{ee} >\right]\frac{G_e e}{\gamma_e} + \lambda\psi_{ie}\left[N_{ie}^{\beta} S_i(h_i^n) + < p_{ie} >\right]\frac{G_i e}{\gamma_i}}{\tau_e}\right] \quad (19)$$

$$\left[\frac{dh_i}{dt}\right]_{drift} = \left[\frac{h_i^{rest} - h_i^n + \psi_{ei}\left[(N_{ei}^{\alpha} + N_{ei}^{\beta})S_e(h_e^n) + < p_{ei} >\right]\frac{G_e e}{\gamma_e} + \lambda\psi_{ii}\left[N_{ii}^{\beta} S_i(h_i^n) + < p_{ii} >\right]\frac{G_i e}{\gamma_i}}{\tau_i}\right] \quad (20)$$

We simulate the continuous time series $\xi(t)$ by an equivalent discrete time series η_n at time values $t_n = n\Delta t$, Δt is the time step, with zero mean and delta variance. The random component of the derivative in simulated form, arguably, is proportional to $\frac{1}{\sqrt{\Delta t}}$ [2]. The continuous noise functions (15) and (16) become the following discrete noise time-series:

$$\Gamma_e^n = \frac{\sqrt{\alpha}}{\tau_e}\left(\frac{\eta_1^n}{\sqrt{\Delta t}}\sqrt{< p_{ee} >}\frac{G_e e \psi_{ee}}{\gamma_e} + \frac{\eta_2^n}{\sqrt{\Delta t}}\sqrt{< p_{ie} >}\frac{G_i e \psi_{ie} \lambda}{\gamma_i}\right) \quad (21)$$

$$\Gamma_i^n = \frac{\sqrt{\alpha}}{\tau_i}\left(\frac{\eta_3^n}{\sqrt{\Delta t}}\sqrt{< p_{ei} >}\frac{G_e e \psi_{ei}}{\gamma_e} + \frac{\eta_4^n}{\sqrt{\Delta t}}\sqrt{< p_{ii} >}\frac{G_i e \psi_{ii} \lambda}{\gamma_i}\right) \quad (22)$$

Δt is selected based on the range of parameters considered here. For too large a time step, the numerical routine is unstable, leading to an incorrect time signal. However, too small a time step may increase the computing complexity. So the largest time step that could be safely used was 0.1ms [2]. The parameters are chosen according to the nomenclature of Steyn-Ross et al.[1]. The parameter values are based on experimentally measured physiological quantities. A more in depth discussion of these equations can be found elsewhere [3-5]. Solving equations (17)-(22), the time series of excitatory and inhibitory populations can be derived. Subtracting h_e^0 from the time-series voltages of excitatory population, the difference is regarded as the simulated EEG.

From the above descriptions we know that the fluctuations of excitatory neurons around steady states are taken as the source of sEEG. It can be seen from Figure A and the above equations that the excitatory neurons and inhibitory neurons influence each other, the voltages of inhibitory neurons affect excitatory neurons by the sigmoidal transfer function $S_i(h_i)$.

1. Steyn-Ross ML, Steyn-Ross DA, Sleigh JW. Modelling general anaesthesia as a first-order phase transition in the cortex. Progress in Biophysics & Molecular Biology. 2004;85(2-3):369–85.
2. Wilcocks LC. Investigations of a Stochastic Model of the Electrical Behaviour of the Cerebral Cortex: University of Waikato; 2001.
3. Liley DT, Cadusch PJ, Wright JJ. A continuum theory of electro-cortical activity. Neurocomputing. 1999;26:795-800.
4. Liley DT, Cadusch PJ, Dafilis MP. A spatially continuous mean field theory of electrocortical activity. Network: Computation in Neural Systems. 2002;13(1):67-113.
5. Bojak I, Liley D, Cadusch P, Cheng K. Electrorhythmogenesis and anaesthesia in a physiological mean field theory. Neurocomputing. 2004;58:1197-202.
